# Interference in early dual-task learning by predatory mites

**DOI:** 10.1016/j.anbehav.2017.09.005

**Published:** 2017-11

**Authors:** Inga C. Christiansen, Peter Schausberger

**Affiliations:** a Group of Arthropod Ecology and Behavior, Department of Crop Sciences, University of Natural Resources and Life Sciences Vienna, Austria; b Department of Behavioural Biology, University of Vienna, Austria

**Keywords:** cannibalism, kin recognition, limited attention, multisignal environment, multitasking, Phytoseiidae, selective attention

## Abstract

Animals are commonly exposed to multiple environmental stimuli, but whether, and under which circumstances, they can attend to multiple stimuli in multitask learning challenges is elusive. Here, we assessed whether simultaneously occurring chemosensory stimuli interfere with each other in a dual-task learning challenge. We exposed predatory mites *Neoseiulus californicus* early in life to either only conspecifics (kin) or simultaneously conspecifics (kin) and food (thrips or pollen), to determine whether presence of food interferes with social familiarization and, vice versa, whether presence of conspecifics interferes with learning the cues of thrips. We found that *N. californicus* can become familiar with kin early in life and use kin recognition later in life to avoid kin cannibalism. However, when the juvenile predators were challenged by multiple stimuli associated with two different learning tasks, that is, when they grew up with conspecifics in the presence of food, they were no longer capable of social familiarization. In contrast, the presence of conspecifics did not compromise the predators' ability to learn the cues of thrips. Memory of experience with thrips allowed shorter attack latencies on thrips and increased oviposition by adult *N. californicus*. Proximately, the stimuli for learning the features of thrips were apparently more salient than those for learning to recognize kin. We argue that, ultimately, learning the cues of thrips at the expense of impeded social familiarization pays off because of negligible cannibalism risk in the presence of abundant food. Our study suggests that stimulus-driven prioritization of learning tasks is in line with the predictions of selective and limited attention theories, and provides a key example of interference in dual-task learning by an arthropod.

Learning is defined as experience-based change in behaviour and memory retention over time (Alcock, 2005; Dukas, 2008; Lincoln, Boxshall, & Clark, 1998), and an omnipresent phenomenon in animals including arthropods (Alloway, 1972; Papaj & Lewis, 1993; Smid & Vet, 2006). The ability to learn allows animals to adjust to changing environments and is largely assumed to have positive effects on evolutionary fitness (Papaj & Lewis, 1993). However, learning is only beneficial to fitness as long as the benefits outweigh the costs (Dukas, 1999; Stephens, 1991). Neuronal activity during learning, that is, collecting, processing and storing information, as well as recalling and connecting different bits of information, requires energy, which is traded off against energy needed for other activities and cognitive tasks. These trade-offs represent the costs of learning (Bernays, 1998; Dukas, 1999; Jaumann, Scudelari, & Naug, 2013; Laughlin, 2001). For example, Mery and Kawecki (2003, 2004, 2005) showed for the fruit fly *Drosophila melanogaster* that an improved learning ability may come at the expense of poorer competitive ability, reduced oviposition rate and shortened longevity, and Snell-Rood, Davidowitz, and Papaj (2011) observed that adult cabbage white butterflies, *Pieris rapae*, had lower oviposition rates when they had to learn to search for a rare host plant type than when they fed exclusively on the common type. Along with physiological trade-offs, learning may incur cognitive trade-offs. Cognitive trade-offs may be, but are not necessarily, linked to energetic deficits caused by energy-demanding learning (Jaumann et al., 2013) and are commonly due to processing limitations of the cognitive system and associated resources. Cognitive trade-offs are especially likely to occur when animals are challenged to simultaneously process multiple unimodal stimuli within the same or across different learning tasks (Cohen, Konkle, Rhee, Nakayama, & Alvarez, 2014; Duncan, Martens, & Ward, 1997).

Animals are usually exposed to multiple, simultaneously occurring stimuli rather than to single stimuli occurring in isolation. Exposure to multiple stimuli does not imply that the animals divide their attention equally among those stimuli; they may rather selectively pay more attention to one stimulus or learning task and neglect the others (Dukas, 2002, 2004; Johnston & Dark, 1986; Lavie, 2005; Wiederman & O'Carroll 2013; Van Swinderen, 2007). Since the perceptual and neuronal systems are limited, it is simply not possible to process every piece of information that is available at a given time. Focusing on one stimulus or task may improve learning, whereas dividing attention to different stimuli may compromise it (Dukas, 1999, 2002). Owing to the interrelated physiological and cognitive benefitecost trade-offs of learning, animals should be selected to flexibly attend to, focus on and learn those stimuli that promise the highest fitness gains, and filter out information of lower relevance. Which stimuli are ultimately more important than others depends on the contexts, tasks and circumstances. Which stimuli prevail over others depends, proximately, also on the predisposition of the sensory system and the salience of the stimuli. Multiple simultaneously occurring stimuli may be associated with different sensory modalities, such as vision, audition, touch or chemosensation, or the same sensory modality. While exposure to, and integration of, multiple multimodal stimuli may lead to cross-modal facilitation of learning and enhancement of memory formation (see, for example, synergistic interactions between olfactory and visual stimuli in learning by *Drosophila* (Guo & Guo, 2005), or Higham and Hebets (2013) for the advantage of multimodal signals in animal communication), exposure to multiple unimodal stimuli may result in interference (Duncan et al., 1997; Cohen et al., 2014; but see Rubi and Stephens (2016) for no difference in learning of multi- and unimodal signals by blue jays, *Cyanocitta cristata*, in the context of communication). Interference among multiple unimodal stimuli (Duncan et al., 1997) may be due to limitation at the perceptual and/or neuronal levels (e.g. Cohen et al., 2014; Pashler & Johnston, 1998).

Interaction between multiple stimuli within the same learning task, such as overshadowing and blocking, is well known from classical conditioning (Mackintosh, 1971). Typical examples of interaction between multiple, simultaneously occurring stimuli across learning tasks are the effects of the presence of conspecifics on learning performance in foraging tasks. The mere presence of conspecifics has frequently been shown to profoundly affect individual learning, in both a negative way, due to distraction, and a positive way, due to social facilitation (Chabaud, Isabel, Kaiser, & Preat, 2009; Zajonc, 1965). For example, the presence of conspecifics may compromise food-learning performance in birds (Klopfer, 1958) but enhance it in sea snails *Aplysia fasciata* (Schwarz & Susswein, 1992). While the effects of the mere presence of conspecifics on foraging learning tasks are widely documented, we are not aware of any study that assessed whether the presence and learning of food cues affects social familiarization, that is, learning the features of conspecifics.

We assessed interference in early dual-task (social and foraging) learning by the predatory mite *Neoseiulus californicus* (Acari: Phytoseiidae). Most animals, including predatory mites, in their early life phases are highly sensitive to environmental stimuli. Experiences made in this phase may have profound and persistent consequences for developmental and behavioural trajectories (e.g. Strodl & Schausberger, 2012, 2013). Phytoseiid mites develop through five life stages (egg, larva, protonymph, deutonymph, adult), are eyeless and primarily use chemo- and mechanosensory modalities (Sabelis & Dicke, 1985). Recent studies revealed that phytoseiid mites, such as *Phytoseiulus persimilis, Amblyseius swirskii* and *N. californicus*, are especially well able to learn chemosensory cues as larvae and early protonymphs, and able to retain memory through several moulting events into adulthood. Early learning of chemosensory cues occurs in social (*P. persimilis*: Schausberger & Croft, 2001; Schausberger, 2007; Rahmani, Hoffmann, Walzer, & Schausberger, 2009; Strodl & Schausberger, 2012, 2013), foraging (*N. californicus*: Schausberger, Walzer, Hoffmann, & Rahmani, 2010; *A. swirskii*: Christiansen, Szin, & Schausberger, 2016) and intraguild predation (*P. persimilis, Amblyseius andersoni*, *N. californicus*: Walzer & Schausberger, 2011) contexts. Learning in social contexts is especially relevant for predatory mites living, at least temporarily, in groups, which are mostly species adapted to exploit patchily distributed spider mites as prey. The focal animal of our study, *N. californicus*, is a generalist predator with a ranked diet preference for spider mites (Castagnoli et al., 2003; Croft, Monetti, & Pratt, 1998; McMurtry & Croft, 1997). The patchy distribution of the spider mites may result in clumped distribution of the predators, allowing frequent mutual encounters among conspecifics but also entailing the risk of cannibalism (Schausberger, 2003). Such conditions promote the evolution of kin recognition abilities (e.g. Fellowes, 1998).

To enhance survival in times of food scarcity and/or to eliminate conspecific competitors, most predatory mites, including *N. californicus*, engage in cannibalism (Walzer & Schausberger, 1999; Schausberger & Croft, 2000; Farazmand, Fathipour, & Kamali, 2014; for review see Schausberger, 2003). Cannibalism is a widespread phenomenon in both vertebrates and invertebrates (Elgar & Crespi, 1992; Fox, 1975) and occurs primarily, or intensifies, when other food is unavailable or of low quality and/or rare (Schausberger, 2003). When both conspecific and heterospecific predatory mites are present, many generalist predatory mite species, including *N. californicus*, use species recognition abilities to preferentially feed on heterospecifics (Schausberger, 2003). Regarding conspecific prey, due to the risk of inclusive fitness loss (Hamilton, 1964), potential cannibals are expected to avoid kin cannibalism when they have other diet options. This has, for example, been shown for *P. persimilis*, which is more strongly adapted to spider mite prey, and which exhibits a stronger tendency to aggregate and live in groups than *N. californicus* (Schausberger, 2007; Strodl & Schausberger, 2012, 2013). In general, kin recognition can be based on contextual cues, recognition alleles and/or prior learning of the features of kin (Mateo, 2004). Among these principal perceptual mechanisms, recognition alleles are extremely rare, presumably because of green beard effects (Dawkins, 1976), whereas kin recognition based on prior learning of the features of kin is most widespread (Mateo, 2004). Accordingly, also in predatory mites such as *P. persimilis, Phytoseiulus macropilis and Iphiseius degenerans*, kin recognition, allowing avoidance of kin cannibalism, is learned and primarily based on prior association (Faraji, Janssen, van Rijn, & Sabelis, 2000; Schausberger & Croft, 2001; Schausberger, 2007). Commonly, juvenile predators become familiar with each other early in life, by direct contact, and later remember and recognize familiar individuals (Schausberger, 2007). *Neoseiulus californicus* has not yet been tested for kin recognition but, like other predatory mites, has a highly sensitive phase early in life, in the facultative-feeding larval stage (Schausberger & Croft, 1999), allowing imprinting on prey: exposure to thrips, which are difficult to grasp, early in life improves foraging on this prey later in life, by shortening attack latencies and increasing predation rates (Schausberger & Peneder, 2017; Schausberger et al., 2010). Depending on food availability, ovipositing *N. californicus* females aggregate or disperse their eggs, with the emerging juvenile mites growing up in the same or different sites (McMurtry & Croft, 1997; Schausberger, 2003; Walzer & Schausberger, 2011). It is not known whether young *N. californicus* are also able to learn in social contexts and, if so, whether the stimuli for learning to recognize particular conspecifics such as kin and those for learning the features of prey interfere with each other. Addressing these questions should at the proximate level provide information about the mites' ability to set priorities in attending to environmental stimuli (selective attention based on stimulus relevance) and/or the relative salience of simultaneously occurring stimuli, and at the ultimate level point to the adaptive significance of showing context-dependent behavioural (developmental) plasticity in foraging and social interactions.

The goals of our research were to investigate (1) whether kin recognition plays a role in cannibalism by *N. californicus*, (2) whether social familiarization and kin recognition by *N. californicus* are compromised when the predators are challenged to simultaneously learn the cues of thrips, and/or (3) whether learning the cues of thrips is compromised by the presence of conspecifics (kin). We hypothesized that (1) *N. californicus* is capable of learning to recognize kin by social familiarization and uses kin recognition to preferentially cannibalize nonkin in choice situations, and (2) exposure to multiple stimuli during the early learning phase, arising from the simultaneous presence of conspecifics and the difficult-to-grasp thrips, compromises their learning performance in either the foraging or social familiarization task, because of interstimuli interference.

## Methods

### Experimental Animals, Population Origins and Rearing

*Neoseiulus californicus* used in the experiments were derived from two populations, hereafter called ‘KO’ and ‘SI’. The KO population was founded with specimens from a mass rearing from the company Koppert BV (Berkel en Rodenrijs, The Netherlands). The SI population was founded with field-collected mites from Trapani, Sicily, Italy (Walzer et al., 2007). Use of two populations allowed us to obtain genetically distant conspecific individuals (nonkin). In the laboratory, the two populations were reared on separate artificial arenas, each consisting of an acrylic tile (15 × 15 cm and 0.2 cm high), resting on a water-saturated foam cuboid in a plastic box (20 × 20 cm and 6 cm high) half-filled with tap water. The edges of the tile were covered with moist tissue paper to prevent the predators from escaping. Additionally, the plastic box containing the rearing arena was placed in a larger plastic tray (45 × 35 cm and 9 cm high), half-filled with tap water containing some drops of dish-washing detergent, and covered by a ventilated transparent acrylic hood. The predators were fed at intervals of 2–3 days by adding bean, *Phaseolus vulgaris*, leaves infested with spider mites, *Tetranychus urticae*, onto the arena. Rearing units were stored in an air-conditioned room at 23 ± 1 °C and 40–60% relative humidity (RH).

The spider mites were reared on whole bean plants, grown at room temperature, 23 ± 2 °C, and 16:8 h light:dark (L:D) photoperiod. Mixed life stages, brushed from infested bean leaves, were used as prey for *N. californicus* females on oviposition arenas and in acrylic cages in experiment 2.

Thrips, *Frankliniella occidentalis*, were reared on green bean pods, in glass jars closed with gauze for ventilation. First-instar thrips were obtained from detached bean leaves (ca. 11 × 13 cm) placed upside down on a solidified 5% agar solution in a closed petri dish (14 cm diameter). For ventilation, a circular opening (1 cm diameter) was cut into the lid and covered with gauze. Adult thrips females were randomly withdrawn from the rearing jar and transferred to the detached bean leaves for oviposition. After 24 h, the thrips females were removed and the petri dishes stored in a climate chamber at 25 ± 1 °C, 65 ± 5% RH and 16:8 h L:D photo-period for 3.5 days. At that time, most larvae had hatched and were singly picked up with a fine red marten's hair brush and placed in an acrylic cage. The acrylic cages, also used in the experiments, consisted of a circular cavity (1.5 cm diameter) laser cut into an acrylic plate (0.3 cm thick) and covered by gauze on the lower side and on the upper side by a microscope slide, held in place by a metal clamp (Schausberger 1997). Cages were cleaned with 75% ethanol before use. For the learning phase of both experiments, first-instar thrips were killed by deep-freezing at −18 °C for at least 2 h and, after thawing, immediately used in the experiments. Dead thrips were offered to facilitate feeding by the juvenile predators and allow reinforcement (Schausberger & Peneder, 2017; Schausberger et al., 2010).

No ethical approval or specific permit was needed for rearing and experimental use of *N. californicus*, *T. urticae* and *F. occidentalis*, which are neither protected nor endangered species.

### Pre-experimental Procedures

To generate sibling egg groups of *N. californicus*, which were needed in both experiments, gravid females from each of the two *N. californicus* strains, KO and SI, were placed singly onto oviposition arenas, each consisting of a spider mite-infested bean leaf placed upside down on moist filter paper covering a water-soaked foam cuboid (7 × 7 cm and 5 cm high), resting in a plastic box (10 × 10 cm and 6 cm high) half-filled with water. The edges of the bean leaf were covered by strips of moist tissue paper to prevent the mites from escaping. The oviposition arenas were stored in a climate chamber at 25 ± 1 °C, 65 ± 5% RH and 16:8 h L:D photo-period. Eggs of the predators were collected every 24 h and placed in acrylic cages. Eggs from the same mother were placed in the same cage, so that only siblings were together, and cages were stored in the fridge at 8 °C to halt egg development. After 4 days of sampling, approximately 5–10 sibling eggs were available per predator mother.

### Experimental Procedures

We conducted two experiments to test for interference in early dual-task learning challenges. Experiment 1 aimed at determining whether *N. californicus* is capable of social familiarization and kin recognition, and, if so, whether social familiarization (learning kin cues) is compromised in a multistimulus environment, that is, by the simultaneous presence of cues of thrips. Experiment 2 aimed at determining whether a multistimulus environment, that is, the simultaneous presence of kin, interferes with the ability of *N. californicus* to learn the cues of thrips early in life. Socially isolated *N. californicus*, that is, in the absence of conspecific stimuli, have repeatedly been shown to learn the cues of thrips (Schausberger & Peneder, 2017; Schausberger et al., 2010). Each experiment consisted of an early learning phase and a behavioural assay (Table 1). In the early learning phase, groups of freshly hatched larvae and early protonymphs, all of which were close kin (siblings), were or were not exposed to food, and thus perceived only kin cues or simultaneously kin and food cues (Table 1). In the behavioural assay of experiment 1, age-advanced (late) protonymphs were tested for memory of familiar kin, and in the behavioural assay of experiment 2, mated predator females were tested for memory of early thrips experience (Table 1).

### Experiment 1: Interference by Food Cues with Learning Kin Cues

For the learning phase (social familiarization), cages containing 5–10 eggs from single females of KO or SI were randomly assigned to one of three treatments, characterized by the type of food added: (1) no food (control), (2) cattail pollen, *Typha angustifolia* (Nutrimite; Biobest, Belgium) (pollen) or (3) four dead first-instar *F. occidentalis* (thrips). All acrylic cages were stored in a climate chamber at 25 ± 1 °C, 65 ± 5% RH and 16:8 h L:D photoperiod and checked twice daily for the developmental status of the predators. Shrivelled thrips carcasses, either sucked out by the predators or dried out, were replenished after 1 day. After 2–2.5 days, the first predator larvae hatched and, after another day, some larvae within each sibling group had moulted to protonymphs. Depending on the treatment, the larvae could contact and become familiar with siblings, and feed on pollen or dead thrips or not. When the first protonymphs appeared, the behavioural assay was started. Before being subjected to the behavioural assay, the larvae were marked with watercolour (Jolly; Brevillier Urban & Sachs, Vienna, Austria) dots on their dorsal shields to allow us to distinguish between familiar and unfamiliar larvae during the experiment (Schausberger & Croft, 2001). The colours used were red and blue, and randomly assigned to familiar/unfamiliar at each replicate. For the behavioural assay, protonymphs from one population were singly placed in a new cage, containing no food, together with a familiar sibling larva and an unfamiliar larva from the other population. To determine kin discrimination and latency until first attack, the cages were checked every 30 min for 3 h and afterwards once per h until the first successful attack, i.e. an attack leading to death, by the protonymph on one of the two larvae had occurred. The experiment lasted until the first successful attack or until one of the two larvae moulted to a protonymph. If neither happened within 8 h of the first day of observation, the cages were stored in the fridge at 8 °C overnight, to stop activity and development of the mites, and taken out from the fridge on the morning of the next day to continue the observation. Each experimental protonymph and larva was used only once, and each of the three treatments was replicated 106–143 times.

### Experiment 2: Interference by Kin Cues with Learning Food Cues

For the learning phase, cages containing 5–10 sibling eggs from single females of KO and SI were randomly assigned to one of two treatments: (1) no food (to generate thrips-naïve predators) or (2) four dead first-instar thrips added as prey (to generate thrips-experienced predators). All cages were checked twice daily for the developmental status of the predators. As in experiment 1, after 2–2.5 days, the first larvae hatched and, after another day, some larvae had moulted to protonymphs. Depending on the treatment, the larvae could contact and possibly become familiar with siblings and/or contact and feed on thrips. When the first protonymphs appeared, they were transferred to a new acrylic cage and fed with mixed stages of spider mites, which do not interfere with the consolidation of memory of early thrips experience (Schausberger & Peneder, 2017; Schausberger et al., 2010), until they reached adulthood. Once the predators were adult, their sex was determined, based on body size (females are approximately twice as large as males), and males were removed from the experiment. Females received a male mate, randomly withdrawn from the rearing unit, in their cage for 20 h. To start the behavioural assay, mated predator females were singly transferred into a new cage containing four living first-instar thrips. To determine latency until first attack and energy allocation, the cages were monitored every 20 min for 3 h for the first successful attack on thrips by the predators and then again after 24 h to determine the number of eggs produced. Each experimental individual was used only once, and each of the four treatments (two populations, thrips experience yes/no) was replicated 19–22 times. All cages were kept in a climate chamber at 25 ± 1 °C, 65 ± 5% RH and 16:8 h L:D photoperiod.

### Statistical Analyses

Statistical analysis was carried out using SPSS 23 (SPSS Inc., Chicago, IL, U.S.A.); all tests were two-tailed. In the first experiment, we used generalized linear models (GLM), first, to compare the propensity to cannibalism (cannibalism yes, no) as affected by treatment (no food, pollen, thrips) (binomial distribution, logit link), and, second, to analyse the cannibalism preference of the protonymphs of the two populations (KO and SI) for the familiar sibling or unfamiliar nonkin larva (binomial distribution, logit link), and their attack latencies (normal distribution, identity link) within each treatment (no food, pollen or thrips during the social familiarization phase). In the second experiment, we used Cox regression to analyse the likelihood and time of attack on thrips by adult *N. californicus* females from the KO and SI populations in relation to whether they experienced thrips early in life or not. A GLM was used to analyse the influence of population origin and thrips experience on the oviposition rate of adult predator females (normal distribution, identity link).

## Results

### Experiment 1: Interference by Food Cues with Learning Kin Cues

**Figure 1:**
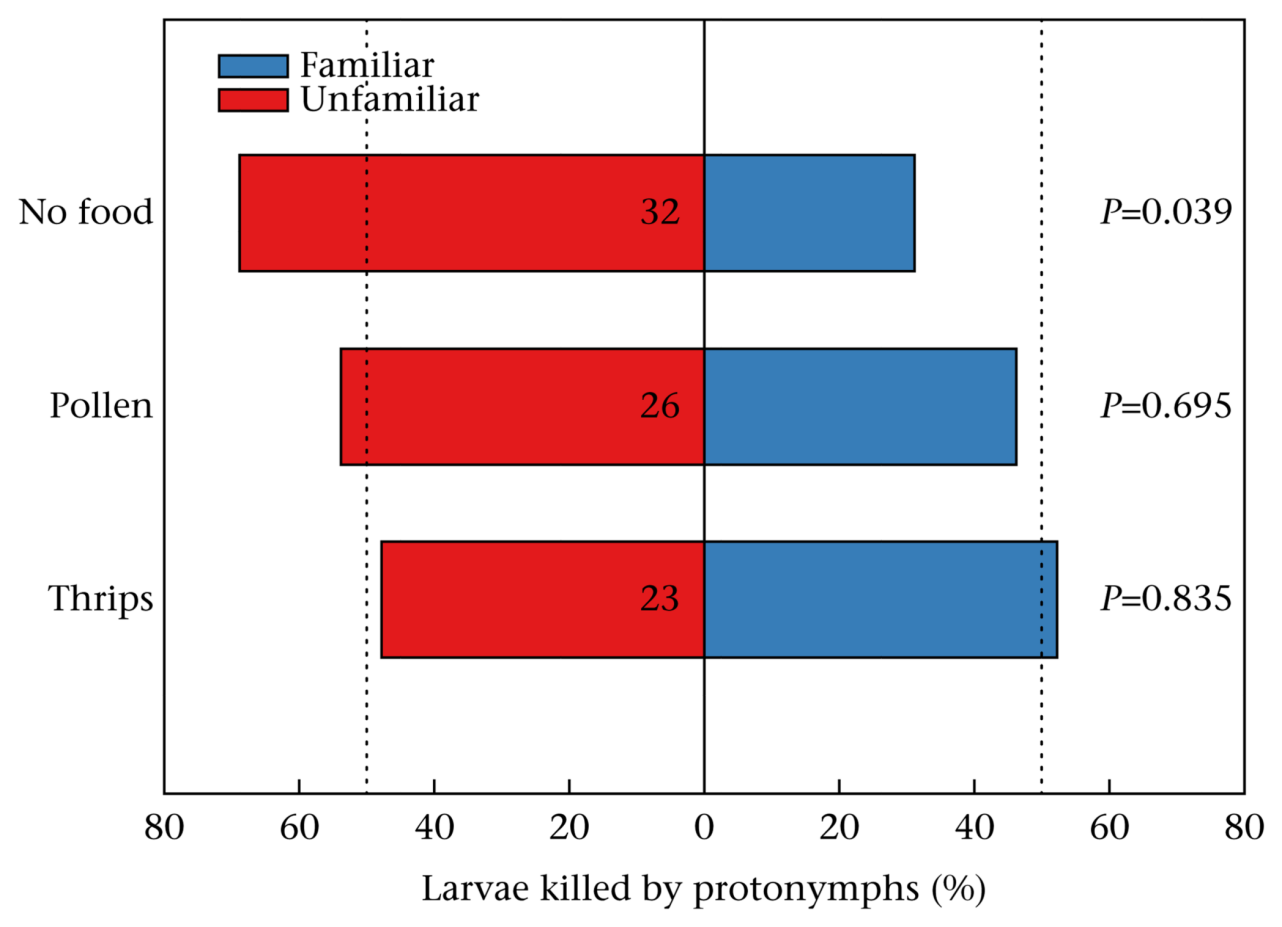
Percentage of larvae first cannibalized by *N. californicus* protonymphs given a choice between an unfamiliar nonkin larva and a familiar sibling larva. During the social familiarization phase, the predators were left without food or additionally given pollen or thrips. Cannibalism was scored at the occurrence of the first successful attack, i.e. attacks leading to death, of the protonymph on one of the two larvae. Numbers inside bars represent the number of replicates; *P* levels (GLM) indicate differences between the attack likelihood on unfamiliar nonkin and familiar sibling larvae within treatments. Vertical dotted lines represent the expected percentages under random choice.

**Figure 2:**
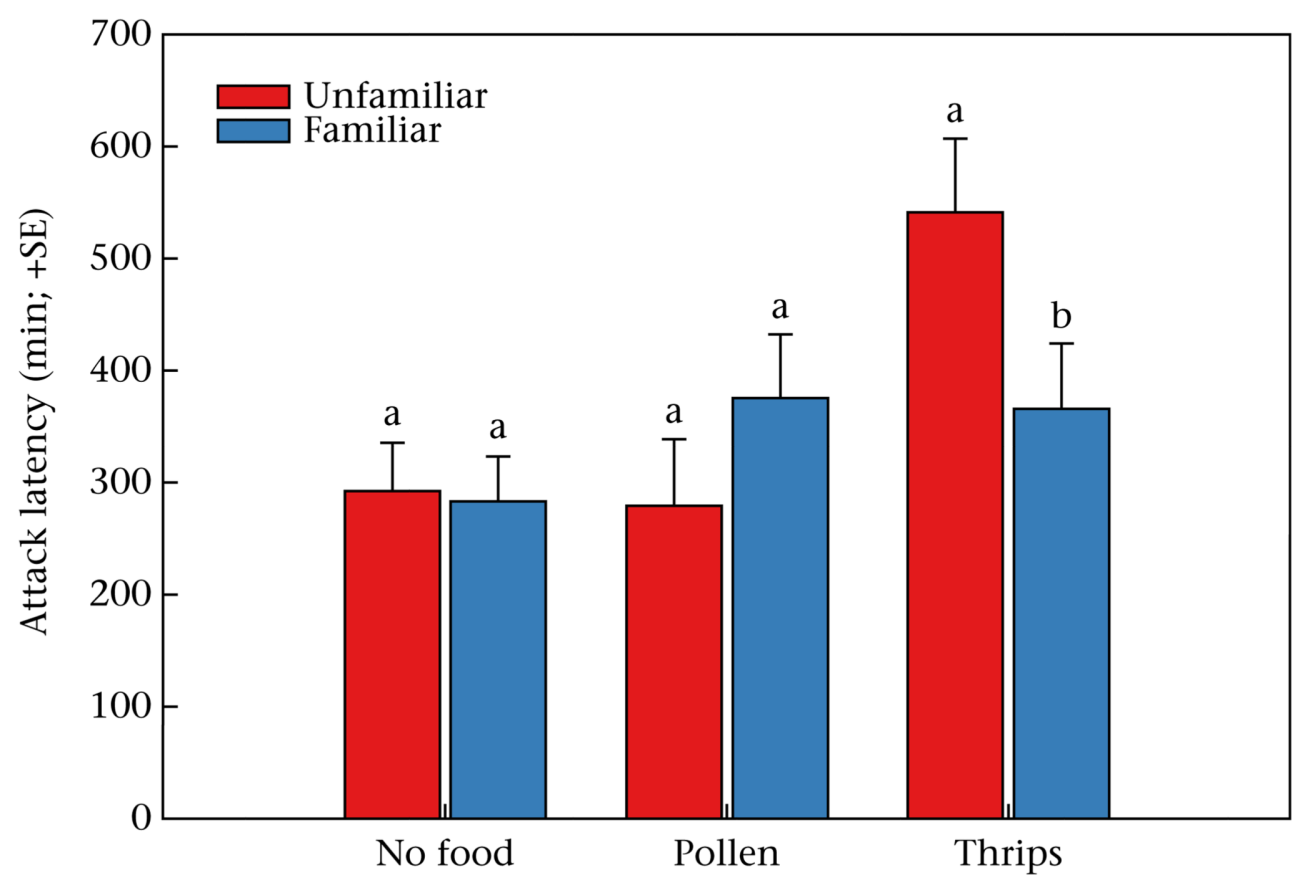
Time elapsed until occurrence of the first successful attack by *N. californicus* protonymphs given a choice between an unfamiliar nonkin larva and a familiar sibling larva as prey. During the social familiarization phase, the predators were left without food or additionally given pollen or thrips. Different lowercase letters on the top of bars indicate significant differences (GLM; *P* < 0.05) between attack latencies on the unfamiliar nonkin and the familiar sibling larva within treatments (pollen, thrips or no food).

Absence (control) or presence of food (thrips or pollen) did not affect the protonymphs' propensity to cannibalism: 20% (no food; 32 of 143), 22% (pollen; 26 of 132) and 23% (thrips prey; 24 of 106) of protonymphs cannibalized a larva during the experimental observation period (Wald $\chi_{2}^{2}=0.400,$ *P* = 0.82). The presence of food, either pollen or first-instar thrips, interfered with social familiarization of the predators during the larval/early protonymphal stage (Fig. 1). When the predator larvae grew up with conspecifics in the absence of food, they could later in life, as protonymphs, discriminate between the familiar sibling and unfamiliar nonkin larvae and preferentially cannibalized the unfamiliar nonkin (GLM: Wald $\chi_{1}^{2}=4.274,$ *P* = 0.04; Fig. 1). In contrast, when the predator larvae grew up with conspecifics in the presence of food, the emerging protonymphs did not distinguish between the familiar sibling and the unfamiliar nonkin larvae, and cannibalized both equally (for pollen: Wald $\chi_{1}^{2}=0.154,$ *P* = 0.70; for thrips: Wald $\chi_{1}^{2}=0.043,$ *P* = 0.84; Fig. 1). In neither treatment (no food, pollen, thrips) did population origin (KO or SI) affect the likelihood of cannibalism of the familiar sibling or unfamiliar nonkin larva (*P* > 0.05 for each treatment). The attack latencies (Fig. 2) of protonymphs on the familiar sibling and the unfamiliar nonkin larvae were similar in the control (GLM: Wald $\chi_{1}^{2}=0.021,$ *P* = 0.89) and pollen treatments (Wald $\chi_{1}^{2}=1.448,$ *P* = 0.23) but differed in the thrips treatment (Wald $\chi_{1}^{2}=4.385,$ *P* = 0.04). Protonymphs that grew up with thrips and conspecifics attacked unfamiliar nonkin larvae later than familiar sibling larvae. In neither treatment (no food, pollen, thrips) did population origin affect the attack latencies of the protonymphs (*P* > 0.05 for each treatment).

### Experiment 2: Interference by Kin Cues with Learning Prey Cues

**Figure 3:**
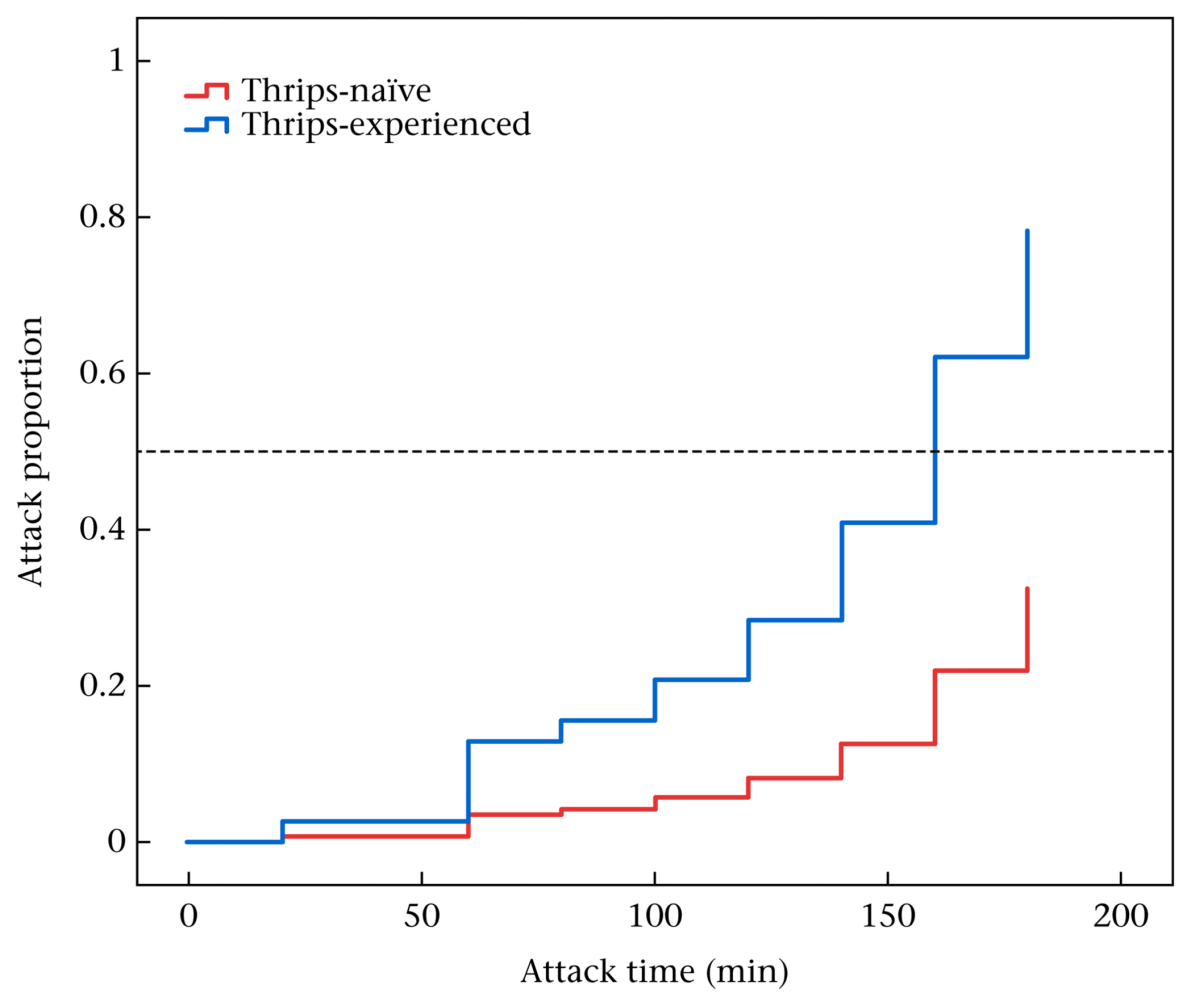
Likelihood and time of attack on first-instar thrips *F. occidentalis* by thrips-naïve and thrips-experienced *N. californicus* females. Thrips experience was established by housing groups of 10 predators in their larval stage in the presence (for thrips-experienced) or absence (for thrips-naïve) of thrips prey. After moulting to protonymphs, both thrips-naïve and thrips-experienced predators were transferred to new cages and fed with mixed stages of spider mites until they reached adulthood. Mated predator females were singly caged with four living first-instar thrips and the cages monitored for the occurrence and time of the first successful attack on thrips.

**Figure 4:**
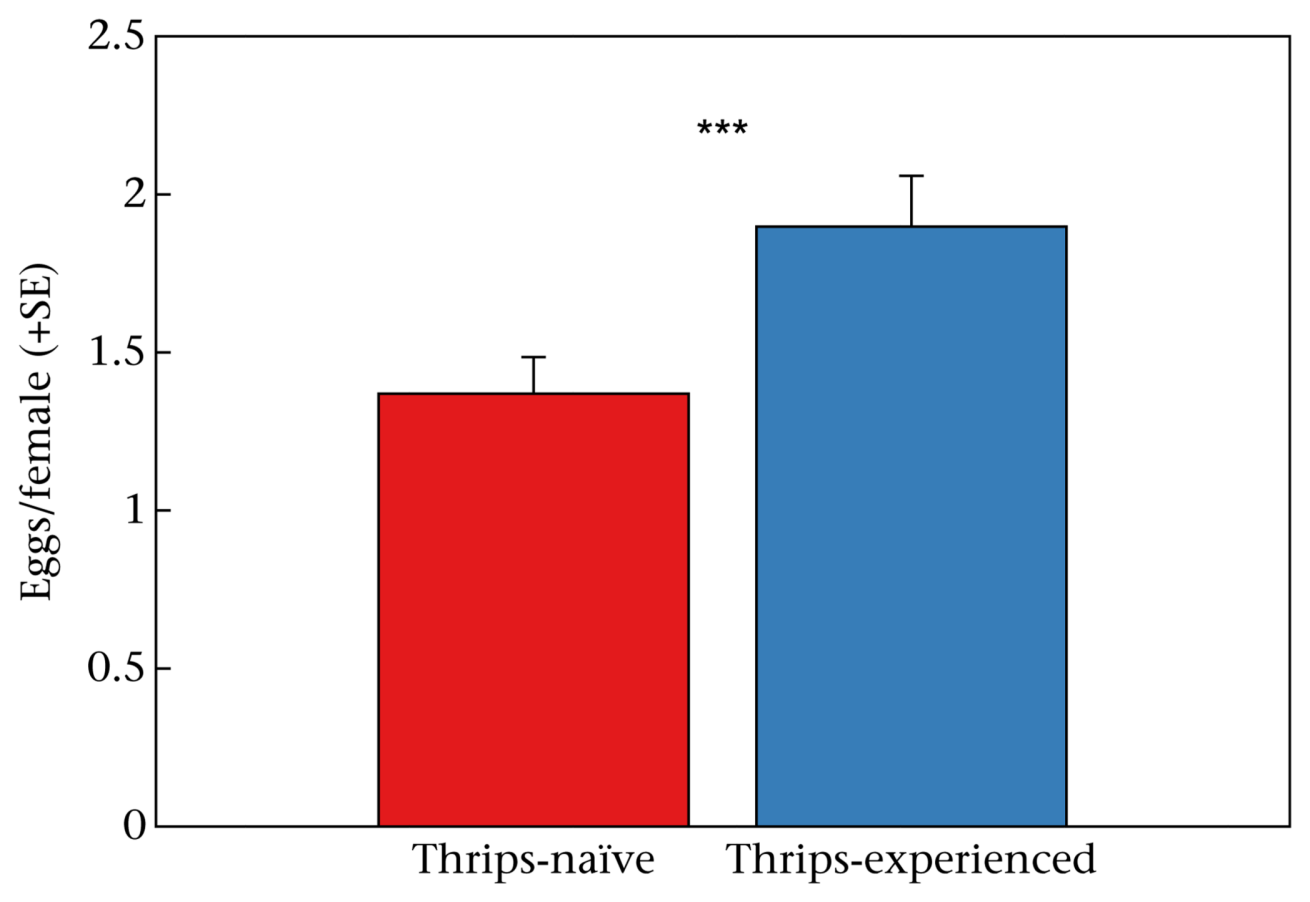
Number of eggs laid by thrips-naïve and thrips-experienced *N. californicus* females. Thrips experience was established by housing groups of 10 predators in their larval stage in the presence (for thrips-experienced) or absence (for thrips-naïve) of thrips prey. After moulting to protonymphs, both thrips-naïve and thrips-experienced predators were transferred to new cages and fed mixed stages of spider mites until they reached adulthood. Mated predator females were singly caged with four living first-instar thrips and oviposition recorded after 24 h. ***P < 0.001; GLM.

The presence of conspecifics (kin cues) did not interfere with learning the cues of thrips. Cox regression revealed that thrips-experienced predators, regardless of their population origin (Wald $\chi_{1}^{2}=0.434,$ *P* = 0.51), were more likely to attack thrips, and did so earlier, than thrips-naïve predators (Wald $\chi_{1}^{2}=16.637,$ *P* < 0.001; Fig. 3). Gravid predator females laid more eggs when they had experienced thrips during early life (GLM: Wald $\chi_{1}^{2}=13.773,$ *P* < 0.001; Fig. 4). Neither population origin (KO or SI) as main effect (Wald $\chi_{1}^{2}=0.064,$ *P* = 0.80) nor the interaction of population origin and thrips experience (Wald $\chi_{1}^{2}=0.049,$ *P* = 0.83) influenced the oviposition rate of the predators.

## Discussion

Our study documents the ability of the predatory mite *N. californicus* to become familiar with kin during the larval and early protonymphal stage, and later recognize kin and preferentially cannibalize nonkin over kin in choice situations. Familiarity-based kin recognition was compromised when the predators were reared in a multistimulus environment, that is, simultaneous presence of conspecifics and food such as pollen or thrips. Grouped larval/protonymphal predators successfully learned the cues of the difficult-to-grasp thrips (experiment 2), but, in presence of thrips, were no longer able to become familiar with kin (experiment 1). Thrips-experienced predators were later in life, as adult females, better able to cope with thrips, which was evident in shorter attack latencies and higher egg production than in thrips-naïve predators. Higher egg production, at the same amount of available prey, indicates higher net energy gains by thrips-experienced than thrips-naïve predators. Overall, our study suggests unidirectional interference in dual-task learning, that is, presence of prey and learning in a foraging task, respectively, interfered with social familiarization but presence of conspecifics (kin) did not interfere with learning in a foraging task.

The first experiment shows that protonymphs of *N. californicus* are able to discriminate between familiar sibling and unfamiliar nonkin larvae and preferentially cannibalize the latter in choice situations. We designed experiment 1 to test for social familiarization, here learning the features of kin, early in life and possible interference by food cues. Addressing the forces selecting for social familiarization was beyond the scope of our study but, presumably, *N. californicus*, like many other animals (e.g. Mateo, 2004), has been selected to use familiarity as a proxy of kin and/or because social familiarity, irrespective of kin, provides direct fitness benefits (Strodl & Schausberger, 2012, 2013). The absence or presence of food (thrips or pollen) during the learning phase, that is, in the larval and early protonymphal stage, did not affect the older protonymphs' propensity to cannibalism, indicating similar motivational, developmental and nutritional states of the protonymphs across treatments. This is probably because of the facultative feeding habit of *N. californicus* larvae, which feed extremely rarely (Schausberger & Croft, 1999). Mere exposure of *N. californicus* larvae to thrips cues, without feeding, is sufficient for learning to occur (Schausberger et al., 2010; Schausberger & Peneder, 2017). Like *N. californicus*, an ability to recognize kin in cannibalism contexts is known from the specialized spider mite predator *P. persimilis* (Schausberger 2001, 2007) and the omnivorous generalist predator *I. degenerans* (Faraji et al., 2000). In these three predatory mite species, *N. californicus, P. persimilis* and *I. degenerans*, ovipositing females aggregate their eggs, allowing frequent encounters among hatching larvae. Grouping and frequent conspecific encounters are considered major preconditions for the evolution of kin recognition abilities (e.g. Fellowes, 1998; Waldman, 1988). *Phytoseiulus persimilis* and, albeit to a lesser degree, *N. californicus* are adapted to exploit patchily distributed spider mite prey (McMurtry & Croft, 1997); *I. degenerans* commutes between flowers, where they feed on pollen, and leaves, where they cluster their eggs (Faraji et al., 2000). Analogous examples of kin recognition and cannibalism come, among others, from butterflies, beetles and salamanders. Larvae of the butterfly *Heliconius erato phyllis* (De Nardin & de Araujo, 2011) and two-spotted ladybirds *Adalia bipunctata* (Agarwala & Dixon, 1993) recognize sibling eggs and preferentially cannibalize eggs from unrelated females. Arizona tiger salamanders, *Ambystoma tigrinum nebulosum*, discriminate not only between siblings and nonkin, but also between different categories of kin, for example siblings and cousins, and preferentially cannibalize the least-related individuals (Pfennig, Sherman, & Collins, 1994). The most plausible ultimate reason why cannibals should preferentially cannibalize unrelated conspecifics when they have a choice is kin selection and inclusive fitness theory (Hamilton, 1964). Under the circumstances tested, killing and feeding on nonkin increases the survival chances of the cannibal and kin, due to nutritional benefits and the elimination of competitors and potential future cannibals (Schausberger & Croft, 2001; Schausberger, 2007).

Kin discrimination by *N. californicus* protonymphs was based on prior familiarization, which occurred only in the treatment without the presence of food cues, and thus the absence of competing chemosensory stimuli, during the larval and early protonymphal stage. In contrast, the second experiment provides evidence that grouping with conspecifics (kin) does not compromise the ability of the predators to learn the cues of thrips. Like predator individuals held in isolation (Schausberger & Peneder, 2017; Schausberger et al., 2010), grouped predators that experienced thrips were, as adult females later in life, more likely to attack thrips, did so earlier and laid more eggs, than thrips-naïve females. Shorter attack latencies correlating with higher oviposition rates, based on early thrips experience, is in line with recent findings on the predatory mite *A. swirskii* (Christiansen et al., 2016; Seiter & Schausberger, 2016).

Proximately, simultaneously perceiving different chemosensory stimuli (cues of kin and thrips), and learning one stimulus (thrips) but not the other (kin), could represent a similar phenomenon to overshadowing in associative learning (Mackintosh, 1971; Schubert, Sandoz, Galizia, & Giurfa, 2015). In overshadowing, a stronger effect of one of two simultaneously present stimuli can be due to differences in salience and/or relevance (Mackintosh, 1971). Duncan et al. (1997) suggested that when several tasks involve two or more different sensory modalities, the learning performance is less affected than when different tasks or sensations are processed via the same sensory modality. In our experiments, the stimuli of thrips and conspecifics were both processed via the chemosensory modality (Schausberger, 2007; Schausberger & Peneder, 2017; Schausberger et al., 2010). Predatory mites sense their environment predominantly by chemo- and mechanosensory receptors (Van Wijk, Wadman, & Sabelis, 2007). For example, they perceive information about distant spider mite patches mainly through olfactory cues emitted by the host plant attacked by the spider mites (e.g. Dicke & Sabelis, 1988; Sabelis & van de Baan, 1983). Similarly, social familiarization by the predatory mite *P. persimilis* is based on volatile and tactile chemosensory stimuli (Muleta & Schausberger, 2013; Schausberger, 2007; Strodl & Schausberger, 2012, 2013). In our experiments, the predators were exposed to multiple stimuli competing for the same chemosensory channel. One likely explanation is that the more salient chemosensory cues of thrips interfered with the perception and processing of the less salient cues of kin. A decreased encounter frequency might be an alternative or additional proximate explanation for lack of social familiarization in the presence of food but is unlikely to be an exclusive explanation because mutual encounter and touching were still observed in treatments with thrips or pollen present.

Thrips cues could simply represent more salient stimuli than kin cues, but, if equally salient and competing, *N. californicus* might have been selected to attend more to stimuli of prey than to those of kin when both are present. Ultimately, learning the cues of kin seems of minor importance for *N. californicus* in environments with abundant food, because the likelihood and intensity of cannibalism is primarily a function of food availability, and less important than learning the cues of a difficult-to-grasp prey such as thrips. Optimizing foraging by learning is highly fitness relevant because it increases survival and reproduction, if current food availability matches future food availability (Christiansen et al., 2016; Seiter & Schausberger, 2016). Owing to the relatively short life cycle of the mites, it is likely that a food source available early in life is still available later in life. Simultaneously present, competing chemosensory cues may thus have, a priori, different priorities for *N. californicus*, making the predators selectively pay more attention to thrips than to conspecifics. Selective and limited attention are the result of information overload, exceeding the processing capacity of the perceptual and/or neural systems (Clark & Dukas, 2003; Cohen et al., 2014; Lavie, 2005; Pashler & Johnston, 1998; Wiederman & O'Carroll 2013; Van Swinderen, 2007). Thus, we argue that, under the circumstances tested, learning the cues of thrips was, for its fitness relevance, of higher priority for *N. californicus* than becoming familiar with kin. Optimizing foraging on thrips provides multiple benefits to the predators, because of the complicated functional roles of thrips interacting with predatory mites. *Frankliniella occidentalis* may feed on predatory and spider mite eggs (Walzer, Paulus, & Schausberger, 2004) and is therefore not only prey but also a potential direct food competitor and intraguild predator of *N. californicus*.

Our findings have relevance for using *N. californicus* in pest management. Revealing that *N. californicus*, a commercially reared biocontrol agent, has capacities for increasing its efficacy against one of the most damaging and difficult-to-control herbivorous pests, i.e. *F. occidentalis*, is of great interest for biocontrol industry and horticulture. The finding that grouped predators may learn prey as well as isolated predators do is important for possible exploitation of target prey conditioning in mass rearing (Christiansen et al., 2016; Schausberger et al., 2010; Seiter & Schausberger, 2016).

## Figures and Tables

**Table 1:** Experimental details of assaying interference by food cues with learning kin cues (experiment 1) and interference by kin cues with learning food cues (experiment 2)

Experiment	Learning phase (5–10 predators/cage)^a^			Behavioural assay (1 predator/cage)	
	
	Life stage	Food	Cues present	Life stage	Prey offered
1	L; early P	No food	Kin	Late P	Conspecific larvae (familiar kin versusunfamiliar nonkin)
		Pollen	Kin and food		
		Thrips	Kin and food		
2	L; early P	No food	Kin	Mated F^b^	Thrips
		Thrips	Kin and food		

Predatory mites *Neoseiulus californicus* were exposed to kin cues with and without food cues early in life and tested for memory of these experiences later in life. L: larva; P: protonymph; F: female.

a All predators within a cage were close kin (siblings).

b From late protonymph to mating, predators were fed on spider mites *T. urticae*.
